# Engineering a probiotic *Bacillus subtilis* for acetaldehyde removal: A *hag* locus integration to robustly express acetaldehyde dehydrogenase

**DOI:** 10.1371/journal.pone.0312457

**Published:** 2024-11-07

**Authors:** Chandler Hassan-Casarez, Valerie Ryan, Bentley M. Shuster, John W. K. Oliver, Zachary D. Abbott

**Affiliations:** ZBiotics Company, San Francisco, CA, United States of America; Gandhi Insititute of Technology and Management, INDIA

## Abstract

We have addressed critical challenges in probiotic design to develop a commercially viable bacterial strain capable of removing the intestinal toxin, acetaldehyde. In this study, we report the engineering of the *hag* locus, a σ^D^-dependent flagellin expression site, as a stable location for robust enzyme production. We demonstrate constitutive gene expression in relevant conditions driven by the endogenous *hag* promoter, following a deletion of the gene encoding a post-translational regulator of σ^D^, FlgM, and a point mutation to abrogate the binding of the translational inhibitor CsrA. Reporter constructs demonstrate activity at the *hag* locus after germination, with a steady increase in heterologous expression throughout outgrowth and vegetative growth. To evaluate the chassis as a spore-based probiotic solution, we identified the physiologically relevant ethanol metabolic pathway and the subsequent accumulation of gut-derived acetaldehyde following alcohol consumption. We integrated a *Cupriavidus necator* aldehyde dehydrogenase gene (*acoD*) into the *hag* locus under the control of the flagellin promoter and observed a rapid reduction in acetaldehyde levels in gut-simulated conditions post-germination. This work demonstrates a promising approach for the development of genetically engineered spore-based probiotics.

## Introduction

Human experiences including sleep [1], weight gain [2, 3], mental health [4, 5], immunity [6, 7], and aging [8, 9] are linked to the intestinal tract and the gut microbiome. The mechanisms underlying these relationships involve a complex interplay between host and microbial proteins and small molecules [10, 11]. While the intestinal tract can be easily influenced by adding food and small molecule drugs, it presents challenges for the *in situ* removal of compounds. Our bodies naturally employ enzymes to clear active small molecules in the intestine. However, the human body and microbiome may lack the necessary enzymes to metabolize molecular byproducts, particularly those we encounter throughout modern life—including the byproducts of processed foods, environmental pollution, reduced sleep, and increased daily stress. Purified enzymes offer a potential solution, but their production and purification can be costly, they can be sensitive to hostile digestive conditions *in vivo*, and they may require a cofactor for function. Engineered probiotics present a promising alternative, enabling *in situ* regeneration of cofactors, protection from proteases within the bacterial cell, and cost-effective manufacturing that minimizes the need for downstream protein purification.

We propose here that active enzyme delivery via engineered probiotics can be successfully achieved through the transient overlay of a non-commensal, endospore-forming, food-safe strain of *Bacillus subtilis*. *B*. *subtilis*, a Gram-positive, rod-shaped bacterial species, is well-regarded for its safety and its ability to form robust endospores (referred to herein as “spores”), facilitating survival in harsh environments [12, 13]. As soil microbes, species of *Bacillus* have been consumed by animals for millions of years, are commonly present in food, and are frequently isolated from human samples [14, 15]. In recent years, recognition of the *Bacillus* genus as a probiotic has expanded with *B*. *coagulans* and *B*. *subtilis* strains such as HU58 and DE111 (now assigned as *B*. *inaquosorum* [16]) becoming widely incorporated as added ingredients into foods and supplements [17–19].

The spore-forming nature of *B*. *subtilis* is not only responsible for its survivability but also its utility as a probiotic, enabling the bacteria to traverse the hostile acidic environment of the stomach. As a matter of product logistics, the stability of spores also facilitates room temperature storage and an extended shelf life. Additionally, vegetative state *B*. *subtilis* cells exhibit well-characterized, strong expression loci, low mutation rates, and a high capacity for enzyme production [20]. Given its extensive history of safe use as a probiotic and its genetic tractability, *B*. *subtilis* is an ideal platform for synthetic biology applications, particularly for the development of engineered probiotics intended for human consumption.

In probiotic design, safety and stability can be enhanced by minimizing edits and the use of exogenous regulatory DNA. Current engineering tools for *B*. *subtilis* include modified promoters, the addition of secretion tags, and methods for scarless transformation using natural competence [21]. To expand the genetic toolkit, we focused on developing a native promoter locus capable of stable, high-level expression with minimal genetic modifications, requiring only a substitution of the gene of interest. Supported by initial work from Dr. Dan Kearn’s lab, which employed a similar approach, we identified the *hag* locus, traditionally associated with the gene encoding flagellin, as a locus capable of highly expressing a non-essential protein during the motile metabolic state of vegetative cells while retaining genomic stability [22, 23]. The *hag* gene possesses a robust endogenous transcriptional promoter, and a ribosome binding site evolved to produce hundreds of thousands of flagellin proteins in a single bacterium [24, 25]. *B*. *subtilis* regulates motility through a sophisticated system of positive and negative regulators, which can switch on and off depending on nutrient availability [26]. In this study, we describe a minimally engineered design that removes two known motility regulators, enabling more robust heterologous protein expression from the *hag* locus. In brief, transcription of *hag* is mediated by the alternative sigma factor, σ^D^, which is post-translationally inhibited by the FlgM protein [27]. Deletion of *flgM* increases σ^D^ activity, leading to higher and more constitutive transcription of the flagellar operon and specifically the *hag* gene. Additionally, the translation of *hag* is controlled by a ribosome binding site that is post-transcriptionally bound and repressed by the CsrA protein [28]. A single point mutation in the CsrA binding site disrupts the protein’s binding and results in more robust translation at the *hag* locus [29].

We leveraged the endogenous promoter of the *hag* locus with a point mutation in the CsrA binding site to generate P_*G->A*_ (henceforth referred to as P_hag*_), and deleted *flgM* to improve transcription through this promoter. As a model application of this expression system *in vivo*, we targeted acetaldehyde (AcA) in the gut, a compound of considerable public interest. After alcohol consumption, the primary pathway for ethanol removal is in the liver, involving its oxidation to AcA by aldehyde dehydrogenase (ADH), followed by its conversion to acetate by acetaldehyde dehydrogenase (ALDH). When ethanol is consumed at a rate that exceeds the liver’s clearance capacity, it recirculates, leading to increased blood alcohol content. Ethanol can equilibrate to the intestine from the bloodstream or reach the colon with food prior to being absorbed. In the case where it reaches the colon, intestinal microbes can convert colonic alcohol into AcA via microbially expressed ADH but cannot not as efficiently convert AcA into acetate. Gut-derived AcA has been shown to increase in a dose dependent manner *in vitro* and will re-enter the bloodstream [30]. Previous studies demonstrate that after alcohol ingestion, the highest levels of AcA are found in the colon *in vivo* [31, 32]. As a point of public interest, AcA is well-documented as a contributor to next-day discomfort associated with alcohol consumption, and its removal has been shown to alleviate this discomfort [33]. Conversely, when the body’s ability to oxidize AcA to acetate is inhibited either chemically (e.g., with disulfiram) or genetically (e.g., single nucleotide polymorphisms in ALDH genes)—amplified discomfort is typically experienced [34, 35].

**Fig 1:**
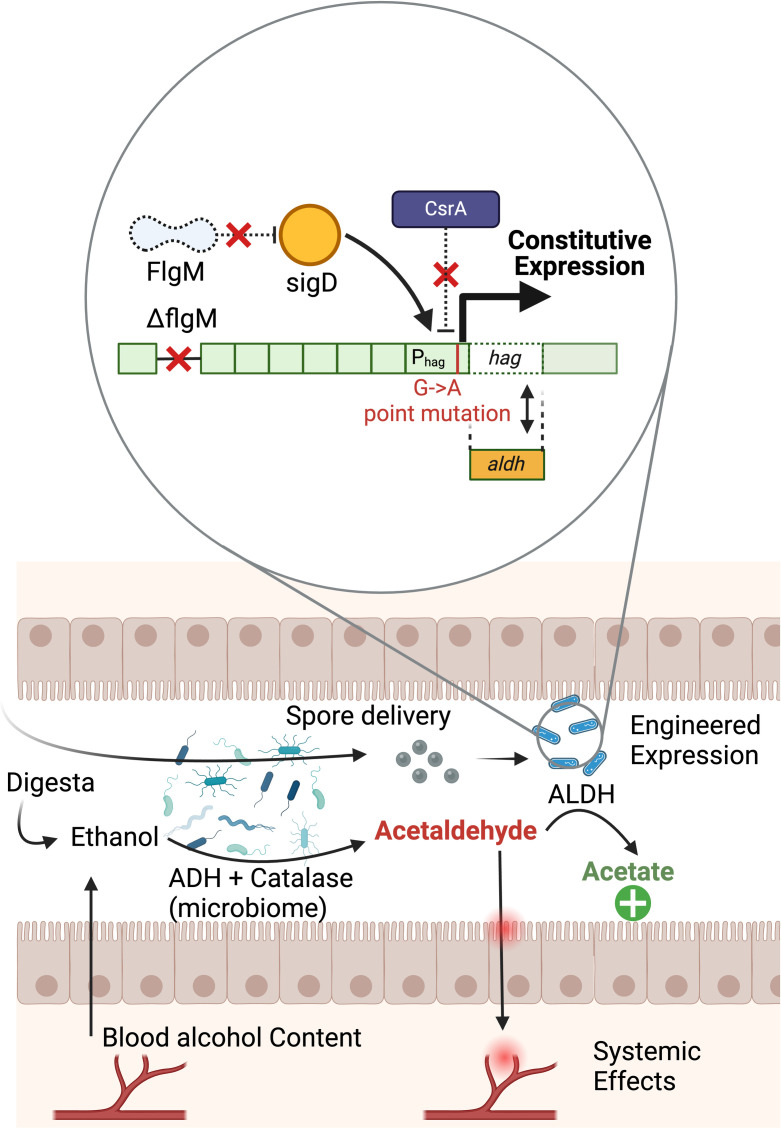
An overview of the engineered promoter and ALDH action in the gut. Top: Optimization of *hag* locus engineering for constitutive and robust heterologous protein expression. Deletion of *flgM* alleviates repression of σ ^D^ for constitutive transcription, while a point mutation in the *hag* promoter prevents CsrA from repressing translation at the *hag* locus. Heterologous expression of ALDH at this site allows the bacteria to catalyze the enzymatic oxidation of acetaldehyde (AcA) into acetate. Bottom: Translational application of engineered chassis for AcA to acetate conversion in the intestine. Ethanol equilibrating from blood alcohol content or reaching the colon directly before absorption into the bloodstream can be converted into AcA by ADH-expressing microbes in the gut microbiome prior to absorption and processing in the liver. AcA can accumulate in the lumen of the gut and subsequently spread throughout the body. Consumption of engineered *Bacillus subtilis* endospores leads to germination and constitutive expression of ALDH, facilitating the conversion of AcA to acetate before buildup and dispersal can occur.

In this study, we present a strain with a genomic integration at the *hag* locus, utilizing an engineered version of its endogenous promoter to drive robust expression of ALDH enzymes, thereby promoting the metabolism of gut-derived AcA (Fig 1). We expressed heterologous *acoD* (Uniprot:P46368), a gene native to *Cupriavidus necator* encoding an enzyme with ALDH activity [36]. Successfully demonstrating the *hag* locus as an effective site for genomic integration enhances the *B*. *subtilis* genetic toolkit and underscores its potential to address real-world challenges through synthetic biology applications. Moreover, by evaluating the heterologous expression post-germination and in simulated intestinal conditions, we can infer the practical utility of this engineered chassis as an *in vivo* protein delivery system. We aim for our findings to establish the *hag* locus as a reliable and versatile site for gene integration, paving the way for further functional exploration in *B*. *subtilis*.

## Results

### The *hag* promoter enables constitutive and robust protein expression

In *B*. *subtilis*, the *hag* locus is an attractive site for genomic integration where it exhibits robust expression of nonessential proteins. To demonstrate its utility, we aimed to engineer a strain of *B*. *subtilis* to express acetaldehyde dehydrogenase (ALDH), enabling the metabolism of gut-derived acetaldehyde. Constitutive expression is ideal to ensure that protein production is not dependent on growth-phase or environmental conditions, which may vary unpredictably within the intestines of a diverse population. To achieve this, we modified the promoter of the *hag* locus to relieve repression due to FlgM and CsrA, resulting in the engineered promoter P_hag*_.

**Fig 2:**
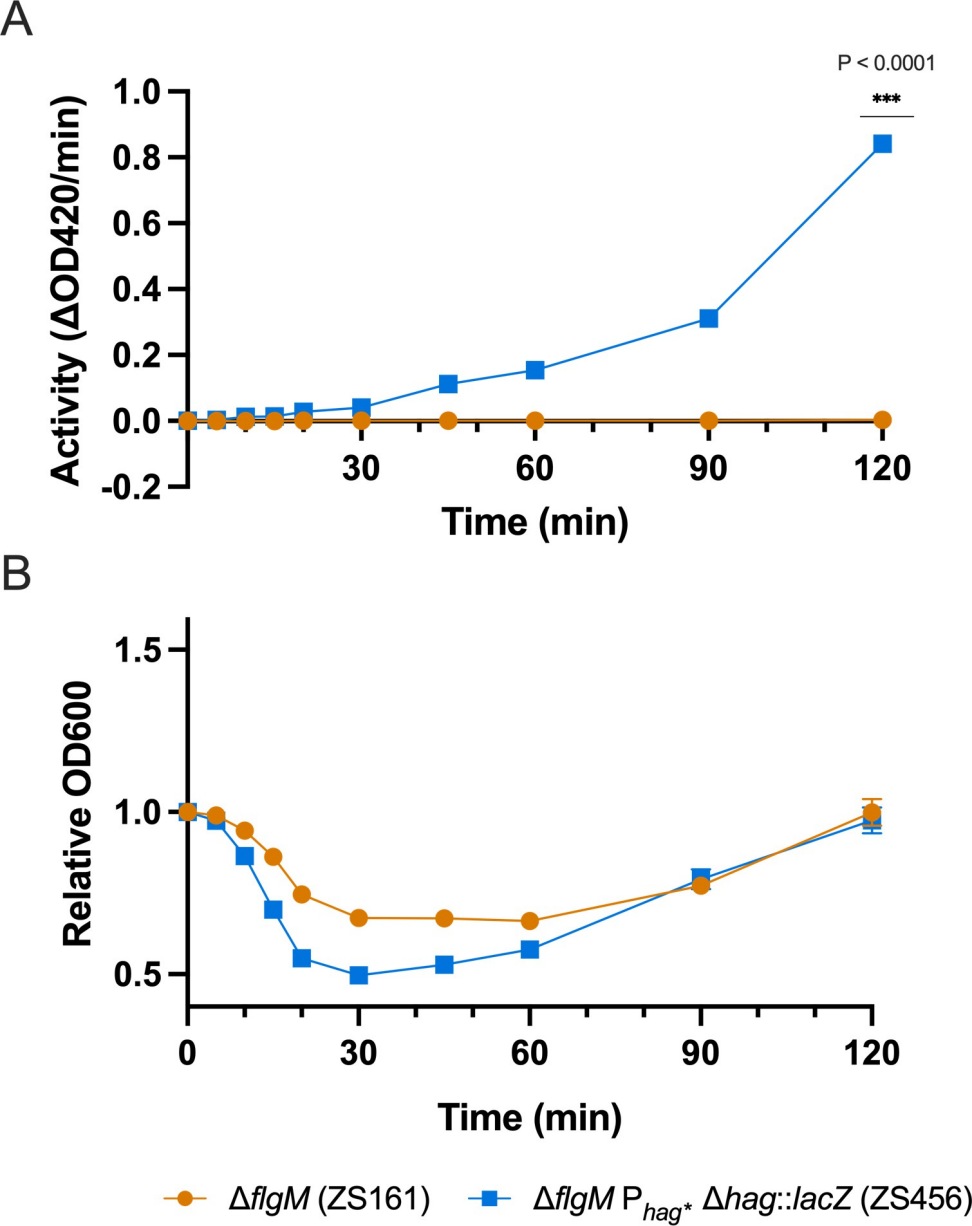
Engineered promoter enables robust protein expression. The efficiency of the P_hag*_ promoter was assessed by quantification of expression in a LacZ reporter strain. (A) LacZ activity of the control strain, ZS161, (orange circles) and the lacZ expression strain, ZS456, (blue squares) was determined at each time point. (B) Germination of ZS161 (orange circles) and ZS456 (blue squares) was evaluated by the change in OD_600_. The data represent the averages from three independent measurements, and error bars represent the standard deviations (SD). If bars are not visible, the SD is smaller than the icon size. Results are representative of 2 experiments.

We assessed expression from the *hag* locus by constructing a LacZ reporter strain, ZS456 (Δ*flgM* P_hag*_ Δ*hag*::*lacZ*), and quantified LacZ activity from germinating cultures. Spores of this strain completed germination by T_30_, as indicated by a drop in absorbance at 600 nm (optical density, OD_600_) (Fig 2B). OD_600_ increased thereafter as the cells entered the outgrowth phase of spore revival. LacZ activity was first detectable at T_45_, consistent with the expected timeline for protein synthesis of nonessential systems during and after germination (Fig 2A) [37]. Activity increased linearly up to T_90_, followed by a sharp rise as outgrowing cells prepared for vegetative growth.

### ALDH activity is robust in the *hag* locus

**Fig 3:**
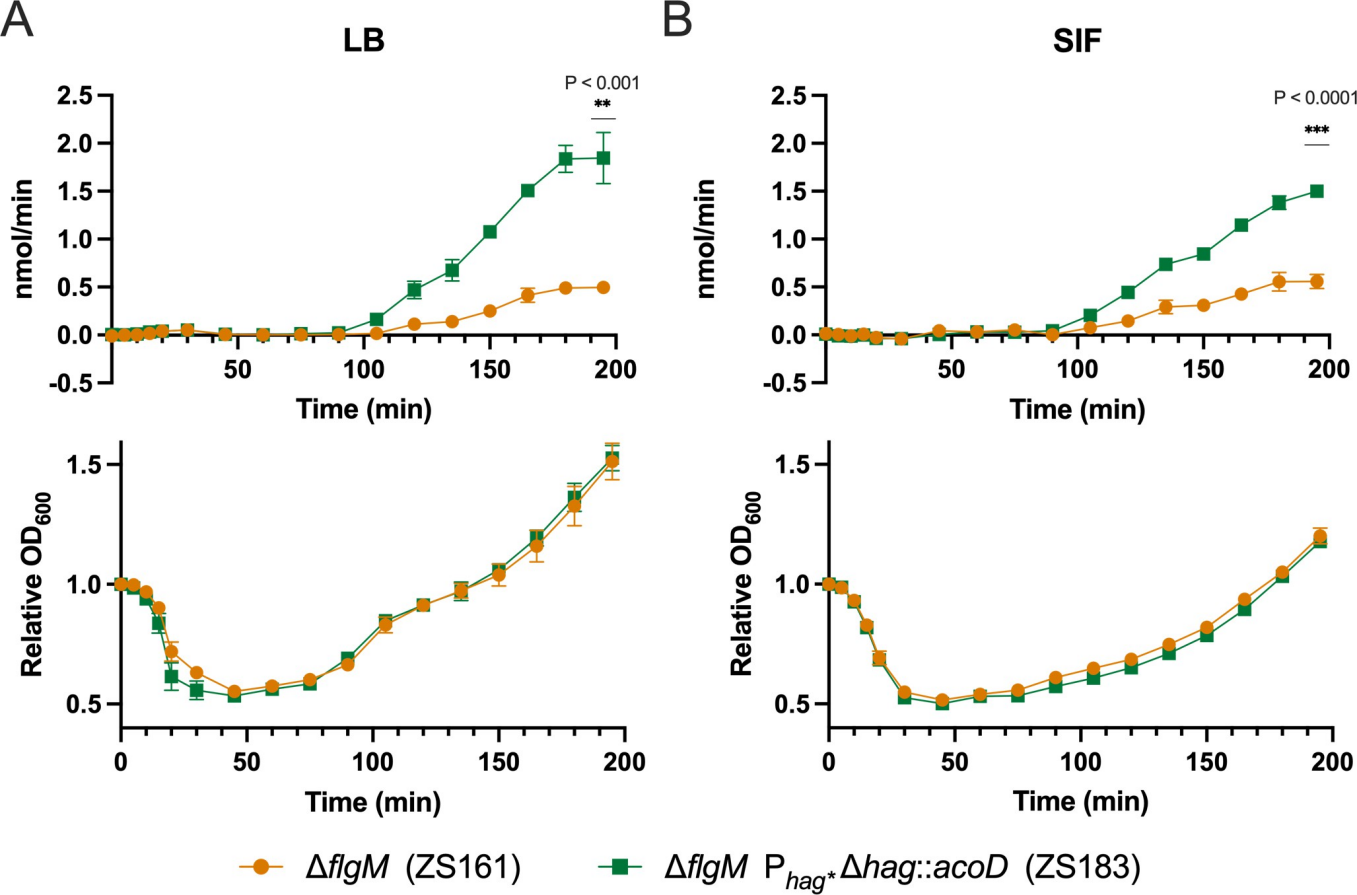
ALDH activity is robust in LB and SIF media. The expression of ALDH was evaluated in nutrient rich (fed state) conditions and in gut simulated media. (A) Top: ALDH activity of ZS161 (orange circles) and ZS183 (green squares) in LB. (B) Top: ALDH activity of ZS161 (orange circles) and ZS183 (green squares) in simulated intestinal fluid (SIF). Bottom: Germination of ZS161 (orange circles) and ZS183 (green squares) in SIF. The data represent the averages from 3 independent measurements, and error bars represent the standard deviations (SD). If bars are not visible, the SD is smaller than the icon size. Results are representative of 2 experiments.

To gauge ALDH activity under the modified *hag* promoter (P_hag*_), we constructed the strain ZS183 (Δ*flgM* P_hag*_ Δ*hag*::*acoD*) and measured activity in germinating cultures in rich media (Fig 3A). Germination was completed by T_45_, as demonstrated by the drop in OD_600_, followed by an increase during outgrowth and subsequent vegetative growth (Fig 3A Bottom). ALDH activity was detected at T_105_ and continued to rise linearly between T_105_ and T_180_, indicative of constitutive expression at this stage of outgrowth (Fig 3A Top).

### ALDH activity is robust after germination in simulated intestinal fluid

To assess whether conditions *in vivo* would impact both germination rates and ALDH activity of the engineered strains, we prepared a simulated small intestinal fluid (SIF) [38]. This media replicates the pH, salt stress, and bile acid stress of the small intestine, where germination is expected to occur. The time to peak germination of spores in SIF was not significantly different from that in rich media (T_45_), but outgrowth and cell division were slightly delayed (Fig 3B Bottom). ALDH expression was detected at T_105_ minutes, approximately one hour after germination, similar to the timing observed in rich media (Fig 3B Top). Activity continued to increase up to T_195_, although the absolute rate of activity was lower than in rich media, consistent with the growth of bacteria in SIF.

### Acetaldehyde removal is robust in whole cells

**Fig 4:**
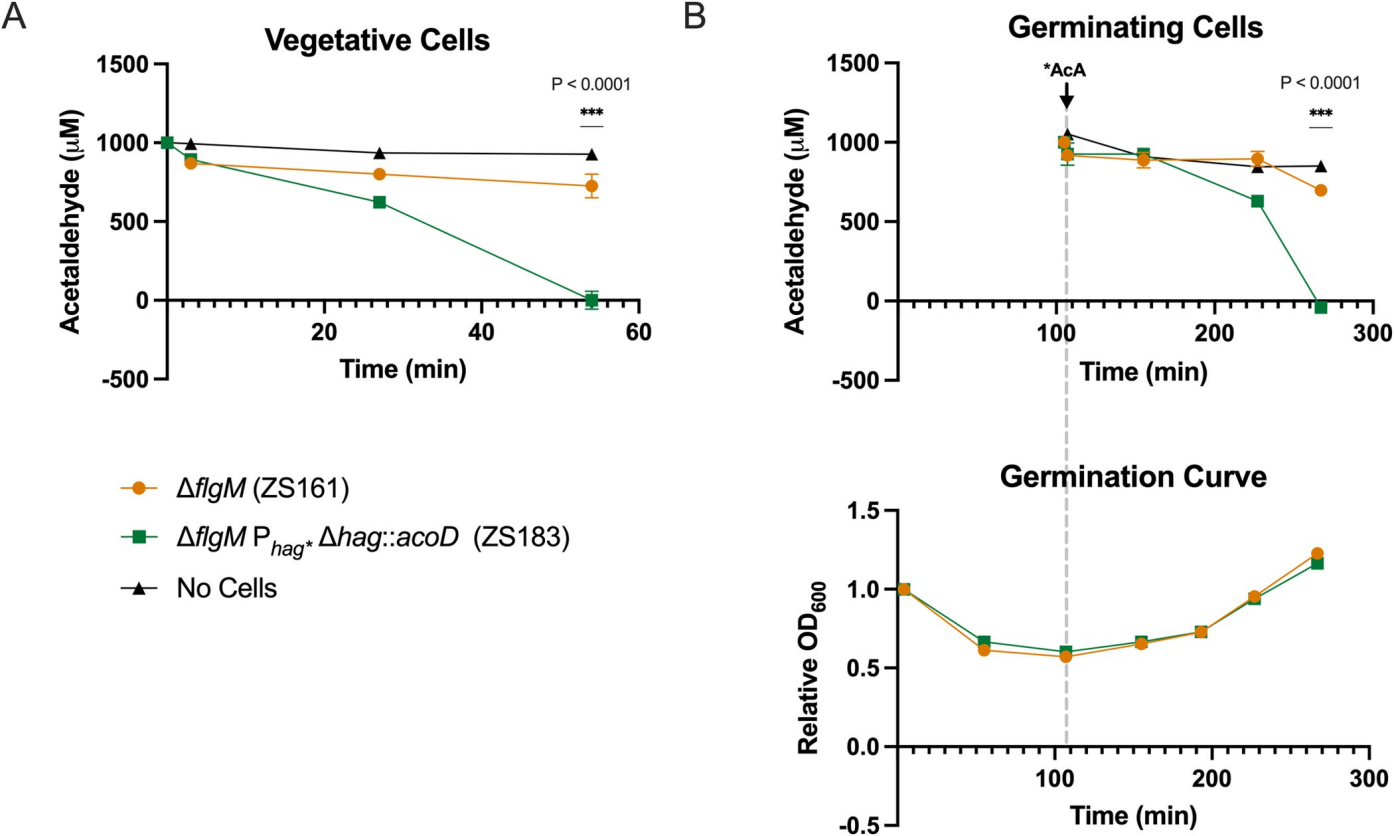
Engineered strain ZS183 efficiently removes acetaldehyde. Acetaldehyde (AcA) removal was quantified in vegetative cells and germinating spores. **A)** AcA removal by vegetative cells of ZS161 (orange circles) and ZS183 (green squares). (B) Top: AcA removal by newly germinated, outgrowing cells of ZS161 (orange circles) and ZS183 (green squares). Bottom: Germination of ZS161 (orange circles) and ZS183 (green squares). Arrow and dotted line indicate when AcA was spiked into the germinating culture. Data represents the average of three independent measurements, and error bars represent the standard deviations (SD). If bars are not visible the SD is smaller than the icon size.

In the previously described experiments, we measured ALDH activity indirectly via the conversion of the enzyme’s cofactor NAD+ to NADH. To directly measure acetaldehyde (AcA) levels, we adapted a protocol by the Ressman lab for high-throughput quantification of AcA [39]. Additionally, unlike previous assays that relied on cell lysis to quantify activity of the P_hag*_ promoter, we conducted the colorimetric assay on vegetative and germinating whole cells. Vegetative cells were grown in LB, rinsed, and added to M9 minimal medium spiked with AcA to a final concentration of 1 mM. We monitored changes in AcA concentration over time by measuring the absorbance at 380 nm in the presence of 2-­amino benzamidoxime (ABAO). Initial AcA levels dropped by 106 μM and 131 μM after exposure to vegetative whole cells of the ALDH-expressing strain ZS183 and the control strain ZS161, respectively, compared to 6 μM in the media-only control, which we attribute to initial AcA interaction with the cells. From T_3_-T_27_, a 271 μM reduction in AcA was recorded in ZS183, 4-fold higher than the 68 μM reduction observed in ZS161 by T_27_ and 8-fold higher by T_54_ (622 μM and 75 μM, respectively). Overall, ZS183 removed more than 99% of AcA, compared to 16.5% for ZS161 (Fig 4A).

### Acetaldehyde removal is active and robust after germination from spores

To determine whether intracellular ALDH activity and NADH turnover are robust in a physiologically relevant timeframe for a probiotic post-germination, the whole-cell AcA removal assay using ABAO was repeated after germination of spores, rather than starting directly with vegetative cells. Spore germination was completed by T_60_ for both strains (Fig 4B Bottom). Due to the reactive nature of AcA, we opted to spike it into the germinating population at T_105,_ just before the cells reached the stage of outgrowth where we previously detected *hag* promoter activity in the ALDH time course (Figs 2 and 3). The concentration of AcA remained unchanged in both cultures up to T_150_, as expected given the expression timeline of σ^D^ dependent genes during germination and outgrowth. By T_220,_ the ALDH-expressing strain ZS183 removed approximately 14-fold more AcA than the control strain ZS161 (296 μM and 21 μM, respectively) and by T_270,_ it removed approximately 4-fold more AcA than the control (925 μM and 219 μM, respectively). Overall, it took 165 minutes for ZS183 to completely deplete the added AcA concentration (Fig 4B Top). The rate of AcA reduction was approximately 41-fold faster in ZS183 than ZS161, 4.52 nmol/mL/min compared to 0.11 nmol/mL/min, respectively.

### Vegetative growth and germination are unaffected by heterologous protein expression in the constitutive *hag* promoter

For *in vivo* enzyme delivery, it is crucial that vegetative cell growth and spore germination are not adversely impacted by the burden of constitutive expression at the *hag* locus, the increased σ^D^ activity due to the *flgM* knockout, or the activity of the expressed enzyme. While we demonstrated *hag* locus activity and spore germination in previous experiments, we additionally screened all strains simultaneously to confirm that there were no defects in growth or germination compared to the parent strain of *B*. *subtilis*, PY79. As expected, we observed no significant changes in growth phases (lag, exponential, stationary) or in germination stages (phase bright, phase dark, germination, ripening, outgrowth) (S1 Fig).

## Discussion

In this study we establish a new robust expression system that relies on derepression of the *hag* locus to achieve high levels of heterologous expression in place of the *hag* gene. Through this system, we engineered a first-of-its-kind acetaldehyde (AcA) degrading probiotic capable of being ingested as a spore. Translational use-cases of a spore-forming probiotic can capitalize on the stability of bacterial spores for oral delivery, convenient storage, and an extended shelf life. However, expression needs to be robust shortly after germination within the intestinal microenvironment. With these considerations in mind, we measured the activity of the P_hag*_ promoter during germination and outgrowth to confirm that robust expression from the *hag* locus occurs in a physiologically relevant timeframe. We detected increasing activity over time, in line with activity profiles of robust gene expression measured shortly after the onset of germination.

We tracked the germination timeline by measuring absorbance at 600 nm. At the timepoints we assessed, changes in OD_600_ are not indicative of increases or decreases in total cell count, but rather they indicate the physiological stages of germination and outgrowth [40]. Consequently, we did not normalize the data based on absorbance as a proxy for overall population density. To ensure activity was attributed to heterologous protein expression rather than population density, we initiated all assays with the equivalent spore OD_600_ for each sample.

Our initial evaluation of the P_hag*_ promoter involved constructing a LacZ reporter strain (ZS456), where we detected activity at T_45_ post-germination initiation. This timing aligns with the expression timelines of early activated cellular processes previously observed during *B*. *subtilis* spore revival (Fig 2A) [37]. In our engineered AcoD-expression strain (ZS183) we observed initial activity at T_105_, nearly an hour later than in the LacZ expression strain (Fig 3). This delay likely reflects a higher detection threshold in the ALDH assay and corresponds with the rise in LacZ activity observed between T_90_-T_120._

Survival of the probiotic chassis and robust *in vivo* germination are essential for achieving enzymatic function within the body. Previous research has demonstrated that *B*. *subtilis* spores can indeed germinate continuously in the small intestines of ileostomy patients, without adverse impacts [17]. Building on this knowledge, we sought to assess the functional activity of our engineered strain under conditions that mimic the gastrointestinal environment. To gauge strain activity in the gastrointestinal tract, we demonstrated germination and ALDH activity in simulated small intestinal fluid (SIF). Spores cultured in both LB and SIF showed robust constitutive expression upon maturation of germinated spores. However, germination and outgrowth proceeded more rapidly in LB compared to SIF, which is likely due to the optimal conditions provided by the rich media (Fig 3).

The ALDH activity experiments were conducted with cell lysates (Fig 3). Therefore, we sought to confirm that similar results could be observed in whole cells, particularly without the addition of the necessary cofactor NAD+. The Δ*flgM* control strain without AcoD (ZS161) demonstrated minimal endogenous AcA dehydrogenase activity, indicated by a slight reduction in AcA compared to wells without whole cells. This observation is likely attributed to non-specific activity of a general aldehyde dehydrogenase or other enzymes with related functions (Fig 4). In both vegetative and germinating AcoD-expressing cells, AcA concentrations rapidly fell below detectable limits, further confirming that ALDH expression under the P_hag*_ is robust and effective for a probiotic designed to remove gut-derived AcA.

## Conclusion

*B*. *subtilis* is an appealing candidate for probiotic applications due to its safety, stability, and ease of genetic manipulation. However, limitations in heterologous protein expression due to a preference for genomic integration, as well as high levels of endogenous protease expression have, lessened the popularity of this organism in the synthetic biology space. For applications where steady expression and stability are favored, integration in *B*. *subtilis* offers a competitive advantage over more common engineering approaches such as lysis circuits, or extrachromosomal plasmids. Additionally, its ability to sporulate offers natural protection and encapsulation, further enhancing its utility as a probiotic. Here, we contribute to the ever-growing engineering toolbox by presenting a novel site of integration with an endogenous promoter, demonstration its potential for real-world applications.

Given that this genetically engineered strain of *B*. *subtilis* is intended for human consumption and will be exposed to a complex intestinal microbiome, we intentionally designed our chassis to minimize the potential for interactions within the gut microenvironment. Introduction of the *acoD* gene from *C*. *necator* is the sole addition of exogenous DNA in ZS183 (Δ*flgM* P_hag*_ Δ*hag*::*acoD*). We specifically selected this gene from a strain that has historically colocalized with *B*. *subtilis* in soil environments. Given the long evolutionary timeline (hundreds of millions of years) that these two species have interacted, and the sheer number of cells that have interacted over that timeline, it is highly probable that *acoD* has been naturally acquired and subsequently lost by *B*. *subtilis* multiple times. Importantly, the function of *acoD* is identical to the function of other endogenous genes in *B*. *subtilis* [41]. Although highly similar to known food-safe genes, the expressed product encoded by *C*. *necator acoD* does not have a well-established history in human food. To alleviate any safety concerns, we conducted a 90-day repeated oral toxicity in rats, which showed no adverse effects on clinical health [42]. The engineered strain was sequenced and confirmed to be free of virulence factors, toxins associated with pathogenicity, and of transferable antibiotic resistance genes, consistent with the known traits of *B*. *subtilis* PY79. Additionally, we conducted tests for hemolysis, antibiotic resistance (minimum inhibitory concentration), and cell cytotoxicity, all of which showed no adverse effects.

The engineered strain ZS183 has since been brought to market (ZB183^TM^)–transparently labeled as “proudly GMO”–to positive public reception. Public adoption of this engineered probiotic, which offers direct consumer benefits, has been strong, with over 5 million bottles sold to date. This aligns with studies of consumer sentiment towards GMOs, which found that while many consumers believe others are opposed to GMOs, they themselves are not averse to products that offer direct benefits [43]. These findings suggest a promising future for the rational engineering of safe and effective probiotics, such as the one demonstrated in this study.

## Materials and methods

### Bacterial strain and growth conditions

All engineered strains described in this work are derived from *Bacillus subtilis* PY79. *B*. *subtilis* strains were routinely cultured at 37˚C with shaking at 250 rpm or grown on agar plates at 37˚C in aerobic environments. Stellar *E*. *coli* HST08 was used for subcloning. *E*. *coli* was cultured at 37˚C with shaking at 250 rpm or grown on agar plates at 37˚C in aerobic environments. Media was supplemented with ampicillin (100 μg/mL) and MLS (25 μg/mL lincomycin and 1 μg/mL erythromycin) when necessary.

### Plasmid construction

To enable expression at the *hag* locus, homologous regions of *hag* were cloned into pMiniMAD which has a temperature-sensitive origin of replication and ampicillin and MLS resistance markers for selection in *E*. *coli* and *B*. *subtilis*, respectively [44]. All plasmids and primers used in this study are listed in Tables 1 and 2, respectively. Briefly, a gBlock (ZP72) containing *GFPmut3* (*gfp*) flanked by 800 bp of upstream and downstream *hag* sequence homology was synthesized by Integrated DNA Technologies (IDT). The 5’ homology region carries a point mutation (G → A) in the *hag* promoter at position -38 relative to the start codon of *hag* to disrupt binding of CsrA (P_hag*_). The gBlock, ZP72 was cloned into pMiniMAD linearized by primers ZP24F and ZP25R to generate pZB149 (P_hag*_ *Δhag*::*gfp*). All subsequent *hag* locus integration vectors were constructed from p149 by replacing GFP with the gene of interest (GOI). To generate pZB163 (P_hag*_ *Δhag*::*acoD*), *acoD* codon optimized to *B*. *subtilis* and synthesized by Genscript was amplified by primers ZP83F and ZP82R and cloned into pZB149 linearized by PCR using primers ZP80F and ZP79R. To generate pZB413 (P_hag*_ *Δhag*::*lacZ*) *lacZ* was amplified using primers ZP203F and ZP204R and cloned into pZB149 linearized by PCR using primers ZP88F and ZP91R. Homologous regions 800 bp upstream and downstream of *flgM* were synthesized by IDT and cloned into pMiniMAD linearized using primers ZP24F and ZP25R to generate pZB147 (Δ*flgM*). All plasmids were confirmed by PCR and Sanger sequencing using primers outlined in Table 2.

**Table 1:** Bacterial strains and plasmids used in this study.

Bacterial Strain or Plasmid	Relevant Background	Source/Reference
**Plasmids**		
pMiniMAD2	oriBsTs amp^r^, er^r^, mls^r^	[44]
pZB147	Δ*flgM*	This study
pZB149	P_hag*_ Δ*hag*::*gfp*	This study
pZB163	P_hag*_ Δ*hag*::*acoD*	This study
pZB413	P_hag*_ Δ*hag*::*lacZ*	This study
**B. subtilis**		
PY79 (ZS6)	*swrA sfpO*	[45]
ZS161	Δ*flgM*	This Study
ZS180	Δ*flgM* P_hag*_ Δ*hag*::*gfp*	This Study
ZS183	Δ*flgM* P_hag*_ Δ*hag*::*acoD*	This Study
ZS456	Δ*flgM* P_hag*_ Δ*hag*::*lacZ*	This Study

**Table 2:** Primers used in the present study.

Primer Name	Sequence 5’-3’	Description
ZP24F	ctggcgttacccaacttaatc	
ZP25R	cttggcgtaatcatggtcatag	Linearize pMiniMAD backbone to generate pZB147 and pZB149
ZP79R	tgttttgttcctccctgaatatgttg	
ZP80F	aagaccttggcgttgc	Linearize pZB149 backbone to generate pZB163
ZP82R	caacgccaaggtcttttttaaaattagaaaaagcccaacgcg	
ZP83F	gggaggaacaaaacaatgaatatggctgaaatcgc	Amplify acoD CDS for generation of pZB163
ZP88F	taattttaaaaaagaccttggcgttg	
ZP91R	cattgttttgttcctccctgaatatgttg	Linearize pZB149 backbone to generate pZB413
ZP95F	gctattgcgtcaaaaccgttc	
ZP96R	caagtgcattcccgcctg	Sanger sequencing of flgM deletion
ZP97F	ctctgaaatgcaccaaattcaattagg	
ZP98R	ggcactcgtcacactaatgttc	Sanger sequencing of all genes inserted in the hag locus
ZP203F	attcagggaggaacaaaacaatgaccatgattacggattc	
ZP204R	cgccaaggtcttttttaaaattatttttgacaccagaccaactgg	Amplify lacZ CDS for generation of pZB413

### Engineered *B*. *subtilis* strain construction

Engineered strains of *B*. *subtilis* were generated by natural competence. Briefly, strains of *B*. *subtilis* were streaked out on LB agar to yield single colonies. Liquid cultures were prepared by inoculating modified competence (MC) media (0.1 M K_3_PO_4_ pH 7.0, 3 mM Na_3_C_6_H_5_O_7_, 3 mM MgSO_4_, 22 mg/mL ferric ammonium citrate, 2% glucose 0.1% casein hydrolysate, 0.2% potassium glutamate) with a single colony and growing to OD_600_ >1.1. From this culture, 400 μL of cells were mixed with >1 μg plasmid DNA and incubated at the restrictive temperature (37˚C) with shaking for approximately 2 hours. Cultures were then plated on agar supplemented with MLS (25 μg/mL lincomycin and 1 μg/mL erythromycin) and incubated overnight at 37˚C. Colonies that grew overnight were assumed to be merodiploids. To stimulate recombination and resolve the merodiploid due to the temperature-sensitive origin of replication in the pMiniMad plasmid, liquid cultures were prepared by inoculating LB broth with a single colony and growing overnight at the permissive temperature (25˚C) with shaking. Overnight cultures were subcultured in fresh LB broth and incubated for 8–12 hours at 25˚C with shaking three times. The final subculture was plated on LB agar and incubated overnight at 37˚C. Single colonies were replica patched on LB agar and LB agar supplemented with MLS. MLS sensitive cured recombinants were PCR-screened for integration and confirmed by Sanger sequencing.

*B*. *subtilis* PY79 was transformed with pZB147 and cured to yield ZS161 (Δ*flgM*). Subsequently ZS161 was transformed with plasmid pZB149 and cured to yield strain ZS180 (Δ*flgM* P_hag*_ *Δhag*::*gfp*). To create the AcoD-expressing strain, ZS161 was transformed and cured of pZB163 to generate ZS183 (Δ*flgm* P_hag*_ Δ*hag*::*acoD*). The LacZ reporter strain was constructed by transformation and curing of ZS161 with pZB413 to generate ZS456 (Δ*flgM* P_hag*_ Δ*hag*::*lacZ*).

### Analysis of vegetative cell growth

Vegetative cell growth was monitored by the change in absorbance at 600 nm (optical density, OD_600_) using a Synergy H1 microplate reader (BioTek; Gen5 v3.10 software). Frozen stocks of *B*. *subtilis* strains were streaked onto LB agar (Lennox; RPI) plates and incubated overnight at 37˚C to yield single colonies. Liquid cultures of *B*. *subtilis* strains were prepared by inoculating LB broth (Lennox; RPI) with a single colony and growing overnight at 37˚C with shaking (250 rpm). The following day, strains were subcultured into fresh LB broth to an OD_600_ = 0.1. Aliquots (200 μL) of each culture were added to a 96-well plate, n = 10. The OD_600_ was measured once every 20 minutes for 8 hours. After background subtraction, OD_600_ values were plotted to generate growth curves.

### Spore preparation and purification

Frozen stocks of *B*. *subtilis* strains were streaked onto nutrient agar (Difco) plates and incubated overnight at 37˚C to yield single colonies. Liquid cultures of *B*. *subtilis* strains were prepared by inoculating nutrient broth (RPI) with a single colony and grown at 37˚C with shaking (250 rpm) until mid-exponential phase of growth. Aliquots (200 μL) from the liquid *B*. *subtilis* cultures were spread onto sporulation media (nutrient agar supplemented with 10% KCl, 1.2% MgSO_4_, 1M Ca(NO_3_)_2_, 10mM MnCl_2_, and 1mM FeSO_4_) and incubated at 37˚C for five days to allow for sporulation. The resulting bacterial lawns were harvested by scraping into ice-cold RO water. The spores were pelleted by centrifugation at 16,000xg for 1 minute. The supernatant was decanted, and the spore pellet was resuspended in RO water. This process was repeated three times.

Spores were purified by density centrifugation through a 20%-50% Histodenz gradient. Following the third wash with RO water, the spore pellet was resuspended in a 20% Histodenz solution, layered on top of the 50% Histodenz solution, and centrifuged at 21,000xg for 5 minutes. After discarding the Histodenz solution, the resulting spore pellet was washed three times by repeated centrifugation (16,000xg, 1 minute) and resuspension in RO water. Purity was assessed by phase contrast microscopy for the appearance of phase bright spores and the absence of vegetative cells. The spores were stored in sterile RO water at 4˚C until needed.

Where indicated, spores were alternatively prepared using a chilled-plate method. In brief, 100 μl of mid log culture was plated on LB agar plates and grown at 37˚C for 72 hours to allow for complete sporulation. Plates were then placed at 4˚C for 4–7 days to allow for natural autolysis by *B*. *subtilis* vegetative cells. Spores were purified from cellular debris by washing in sterile RO water and pelleting by centrifugation at 16,000xg for 2 minutes for at least 3 washes. Purity was confirmed by microscopy for absence of vegetative cells.

### Analysis of spore germination

Spore germination was monitored by the decrease in absorbance at 600 nm (optical density, OD_600_) using an Epoch microplate reader (BioTek, Gen5 v3.10 software). Aliquots of purified spores were suspended in sterile RO water to OD_600_ = 1.0 and concentrated 10X. Germination assays were carried out in 96-well plates at a 360 μL/well final volume. Germination reactions were performed in triplicate wells and consisted of 324 μL LB supplemented with 10 mM alanine and 36 μL of the 10X spore suspension. The initial OD_600_ of the reaction was ~1.0. The OD_600_ was measured once every minute for two hours and normalized using the OD_600_ obtained at time zero [relative OD_600_ = OD_600_(t)/OD_600_(t_0_)]. Resulting curves were analyzed for three metrics: peak rate of percent change in OD_600_ (r_max), time to reach peak rate (t_max), and overall percent drop in OD_600_ (%dOD). An up to 60% drop in OD_600_ can be interpreted as effective germination [40].

### Analysis of LacZ activity

LacZ expression during spore outgrowth was measured in cell lysates by the change in absorbance at 420 nm following the conversion of ONPG to ONP using an Epoch microplate reader (BioTek; Gen5 v3.10 software). Reagents were prepared as follows: assay buffer (0.06 M Na_2_HPO_4_⋅7H_2_O, 0.04 M NaH_2_PO_4_⋅H_2_O, 10 mM KCl, 1 mM MgSO_4_, pH 7.0); BugBuster Lysozyme (BBL) solution (99% BugBuster Protein Extraction reagent (Millipore) and 1% egg white lysozyme (GoldBio) solution (20 mg/mL in 50 mM Tris, pH 8.8)); lysis buffer (90% assay buffer and 10% BBL); ONPG solution (4 mg/mL).

Aliquots of purified spores were suspended in sterile RO water to OD_600_ = 1.0 and concentrated 10X. Flasks containing LB broth were inoculated with 10X spore suspensions to OD_600_ 1.2 and incubated at 37°C with shaking (250 rpm). At time zero (T_0_), 1 mL of the culture was removed and transferred to a chilled microtube on ice. From this sample, 180 μL was transferred to a microplate well containing 180 μL LB, mixed by pipetting, and its OD_600_ measured. The remaining cells were pelleted by centrifugation at 16,000xg for 1 minute, the media was decanted, and the pellet stored at -80°C until needed. These steps were repeated at each time point.

Frozen pellets were resuspended 1:1 in the lysis buffer and incubated at room temperature for 10 minutes. LacZ activity was measured in 96-well plates. Reactions consisted of 60 μL of assay buffer and 100 μL of cell suspension. Absorbance was first measured at 420 nm and 550 nm. Next, 50 μL of the ONPG solution was added to each well. Absorbance readings at 420 nm and 550 nm were then taken every minute for 10 minutes. Light scattering at 420 nm due to cell debris was corrected using the formula: Abs420-(1.75*Abs550). Activity was then calculated as the rate over the linear range with final units of ΔAbs420/min.

### Analysis of ALDH expression in complex media

ALDH expression during spore outgrowth in complex media was measured in cell lysates by the change in absorbance at 340 nm following the production of NADH from NAD+ in the presence of AcA using an Epoch microplate reader (BioTek; Gen5 v3.10 software). Reagents were prepared as follows: 50 mM Tris buffer (pH 8.8); 100 mM NAD+ (in 50 mM Tris buffer, pH 8.8); 50 mM AcA (in RO water); BugBuster Lysozyme (BBL) solution (99% BugBuster Protein Extraction reagent (Millipore) and 1% egg white lysozyme (GoldBio) solution (20 mg/ml in 50 mM Tris, pH 8.8)).

Aliquots of purified spores were suspended in sterile RO water to OD_600_ = 1.0 and concentrated 10X. Flasks containing LB broth were inoculated with 10X spore suspensions to OD_600_ 1.2 and placed in a shaking incubator (250 rpm) at 37°C. At time zero (T_0_), 1 mL of the culture was removed and transferred to a chilled microtube on ice. From this sample, 180 μL was transferred to a microplate well containing 180 μL LB, mixed by pipetting, and its OD_600_ measured and normalized using the OD_600_ obtained at time zero [relative OD_600_ = OD_600_(t)/OD_600_(t_0_)]. The remaining cells were pelleted by centrifugation at 16,000xg for 1 minute, the media was decanted, and the pellet stored at -80°C until needed. These steps were repeated at each time point.

Frozen pellets were resuspended in 200 μL BBL and incubated at room temperature for 10 minutes. Cells were pelleted by centrifugation at 16,000xg for one minute and the lysate transferred to a chilled microtube on ice. Assays were carried out in 96-well plates at a 360 μL/well final volume. Each well was prepared with 264 μL Tris buffer, 36 μL NAD+, and 50 μL cell lysate. Reactions were initiated by the addition of 10 μL AcA. Absorbance at 340 nm was measured every minute for 10 minutes. The peak rate averaged over 4 minutes was then converted to nmol/min AcA using the following equation and a 1:1 ratio of NADH to AcA $\left(nmol/\min=\frac{\Delta OD_{340}}{\varepsilon l}*v*1000\right)$ where ΔOD_340_ is the change in OD_340_ per minute, ε is the extinction coefficient for NADH (6220 M^-1^cm^-1^), *l* is the path length (1 cm), and *ν* is the reaction volume (360 μL).

### Analysis of ALDH activity in simulated intestinal media

ALDH expression during spore outgrowth in simulated intestinal media (16.5 g/L tryptone (RPI) supplemented with FaSSIF Buffer Concentrate (Biorelevant; 3 mM sodium taurocholate, 0.75 mM phospholipids, 148 mM sodium, 106 mM chloride, 29 mM phosphate, pH 6.5)) was measured in cell lysates as described above for ALDH activity in complex media.

### Analysis of acetaldehyde removal by vegetative cells

Acetaldehyde (AcA) removal by vegetative cells was measured by sampling supernatant and assaying AcA using the change in absorbance at 380 nm in the presence of 2-­amino benzamidoxime (ABAO), (as inspired by aldehyde quantifications by [39]) using an Epoch microplate reader (BioTek; Gen5 v3.10 software). Reagents were prepared as follows: M9 minimal media (0.24 M Na_2_HPO_4_•7H_2_O; 0.11 M KH_2_PO_4_; 0.043 M NaCl; 0.093 M NH_4_Cl); sodium acetate buffer (100 mM, pH 4.5); ABAO solution (2.5 mM in sodium acetate buffer)

Frozen stocks of *B*. *subtilis* strains were streaked onto LB agar plates and incubated overnight at 37˚C to yield single colonies. Liquid cultures of *B*. *subtilis* strains were prepared by inoculating LB broth with a single colony and growing at 37˚C with shaking for 6.5 hours. The cells were pelleted by centrifugation at 3,200xg for 10 minutes, the media was decanted, and the pellet washed twice with M9 media. Next, cells were suspended in M9 media to OD_600_ = 1.400 ± 0.050. The change of media was necessary to decrease background interference.

To further account for background interference, each sample was split into two cultures of equal volume. AcA was added to one culture at final concentration of 1 mM, sterile RO water was added to the other culture. Cultures were then placed in an incubator at 30˚C with shaking at 250 rpm. At each time point, an aliquot (200 μL) was removed from each culture and pelleted by centrifugation at 16,000xg for 2 minutes. The supernatant was then transferred to a chilled microtube on ice and immediately quantified in an ABAO assay.

ABAO assays were carried out in 96-well plates at a 350 μL/well final volume. Reactions consisted of 150 μL sodium acetate buffer and 150 μL sample supernatant. A standard curve from 1 mM to 62.5 μM of AcA in M9 media was included on each plate. An initial absorbance measurement at 380 nm was taken. Next, 50 μL of ABAO solution was added to each well. Subsequently, the absorbance at 380 nm was measured once every minute for 20 minutes. In all cases the value at T_20_ was used as final absorbance values for calculations.

Concentrations of AcA were calculated by first subtracting the initial absorbance (380 nm in all cases) of each sample from the final absorbance values for each sample. Next, the absorbance of samples without AcA was subtracted from the absorbance of samples with AcA. For the standard curve a media only well was subtracted from the rest of the curve. The remaining absorbance is attributed to the level of AcA in the sample and the concentration was determined using the internal standard curve. A small background reaction wherein AcA reduces ABAO background reactions was unable to be accounted for and causes some results to drop slightly below zero (the control in which AcA was not added has higher background absorbance than the identical reaction in which AcA has been added and then enzymatically removed). For transparency we have chosen to present these negative values ‘as is’.

### Analysis of acetaldehyde removal by outgrowing cells

Acetaldehyde (AcA) removal during spore outgrowth was measured by sampling supernatant and assaying AcA using the change in absorbance at 380 nm in the presence of ABAO using an Epoch microplate reader (BioTek; Gen5 v3.10 software). Liquid cultures of each sample were prepared by inoculating LB broth (supplemented with 10 mM alanine) with spores prepared by the chilled plate method to OD_600_ = 1.1 ± 0.05. Each sample was then split into two cultures of equal volume and incubated at 37°C with shaking (250 rpm). At T_105_ AcA was added to one culture to a final concentration of 1mM; an equal volume of sterile RO water was added to the other culture. This was repeated for all samples. At each time point, an aliquot was removed from each culture. OD_600_ was recorded and the remaining sample volume was pelleted by centrifugation at 16,000xg for 2 minutes. ABAO assays were performed on the supernatant and AcA concentrations calculated as described above. To calculate rates, the amount of AcA removed between T_107_ and T_267_ was divided by the time between those two points (150 minutes). To account for the effect of evaporation, the rate of AcA lost in the media-only control over the same time was subtracted from the rates in samples containing cells. The remaining rates were used to quantify the effects of the cells in each sample.

## Supporting information

S1 Fig Growth and germination of strains used in this study. The germination and growth profiles of the parent and engineered strains were evaluated by changes in OD600. (A) Growth curve of the wildtype parent strain PY79 (black circles) compared to the engineered strains. The data represent the averages from at least three independent measurements, and error bars represent the standard deviations (SD). If bars are not visible the SD is smaller than the icon size. (B) Germination of PY79 (black circles) compared to the engineered strains. The data represent the averages from at least three independent measurements, and shaded areas represent SD.
(PDF)

S2 Fig Germination of strains in SIF media. Germination at room temperature of ZS161 (orange line) and ZS183 (green line) in SIF was evaluated by changes in OD600 of 360 μl cultures, using an Epoch microplate reader (BioTek; Gen5 v3.10 software). The data represent the averages from three independent measurements, and the shaded area represents the standard deviations (SD).
(PDF)

S1 Table Germination metrics in all strains. (PDF)

S2 Table Germination metrics in SIF vs LB. (PDF)
