# Supplementary material for: Engineering a probiotic Bacillus subtilis for acetaldehyde removal: A hag locus integration to robustly express acetaldehyde dehydrogenase
Source: PLoS One. 2024 Nov 7;19(11):e0312457. doi: 10.1371/journal.pone.0312457 (PMC11542774; doi:10.1371/journal.pone.0312457)
Supplement: S1 Fig — The germination and growth profiles of the parent and engineered strains were evaluated by changes in OD600. (A) Growth curve of the wildtype parent strain PY79 (black circles) compared to the engineered strains. The data represent the averages from at least three independent measurements, and error bars represent the standard deviations (SD). If bars are not visible the SD is smaller than the icon size. (B) Germination of PY79 (black circles) compared to the engineered strains. The data represent the averages from at least three independent measurements, and shaded areas represent SD. (PDF) [file pone.0312457.s001.pdf]

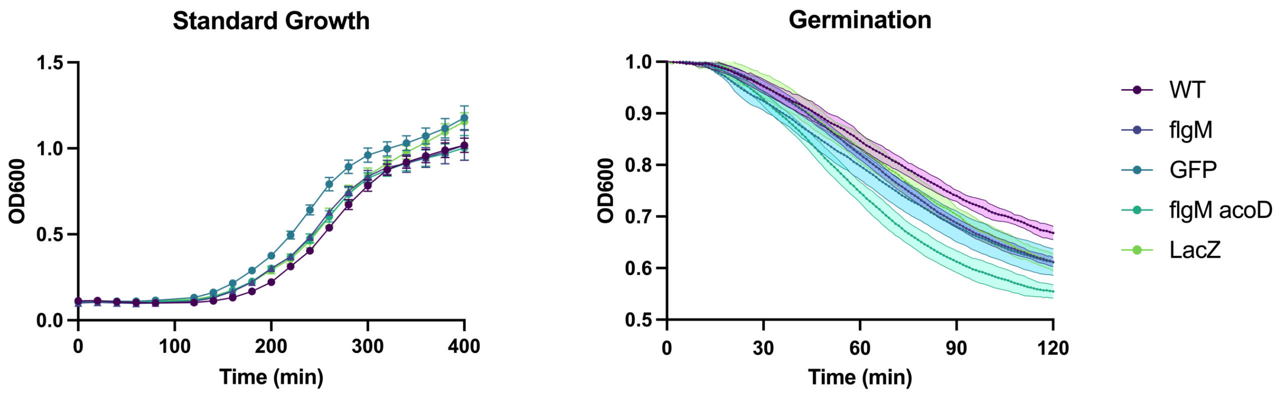

S1 Fig. Growth and germination of strains used in this study. The germination and growth profiles of the parent and engineered strains were evaluated by changes in OD_{600}. (A) Growth curve of the wildtype parent strain PY79 (black circles) compared to the engineered strains. The data represent the averages from at least three independent measurements, and error bars represent the standard deviations (SD). If bars are not visible the SD is smaller than the icon size. (B) Germination of PY79 (black circles) compared to the engineered strains. The data represent the averages from at least three independent measurements, and shaded areas represent SD.
