# Supplementary material for: Engineering a probiotic Bacillus subtilis for acetaldehyde removal: A hag locus integration to robustly express acetaldehyde dehydrogenase
Source: PLoS One. 2024 Nov 7;19(11):e0312457. doi: 10.1371/journal.pone.0312457 (PMC11542774; doi:10.1371/journal.pone.0312457)
Supplement: S2 Fig — Germination at room temperature of ZS161 (orange line) and ZS183 (green line) in SIF was evaluated by changes in OD600 of 360 μl cultures, using an Epoch microplate reader (BioTek; Gen5 v3.10 software). The data represent the averages from three independent measurements, and the shaded area represents the standard deviations (SD). (PDF) [file pone.0312457.s002.pdf]

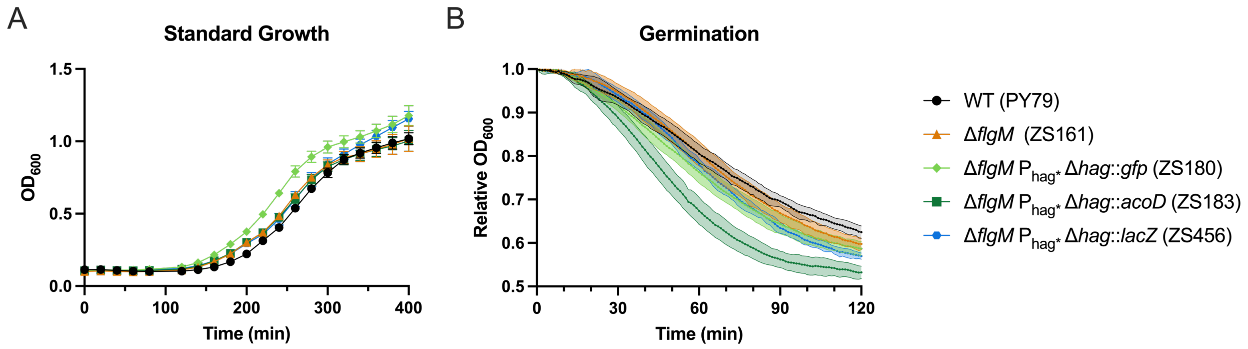

S2 Fig. Germination of strains in SIF media. Germination at room temperature of ZS161 (orange line) and ZS183 (green line) in SIF was evaluated by changes in OD_{600} of 360 µl cultures, using an Epoch microplate reader (BioTek; Gen5 v3.10 software). The data represent the averages from three independent measurements, and the shaded area represents the standard deviations (SD).
