# Supplementary material for: Engineering a probiotic Bacillus subtilis for acetaldehyde removal: A hag locus integration to robustly express acetaldehyde dehydrogenase
Source: PLoS One. 2024 Nov 7;19(11):e0312457. doi: 10.1371/journal.pone.0312457 (PMC11542774; doi:10.1371/journal.pone.0312457)
Supplement: S1 Table — (PDF) [file pone.0312457.s003.pdf]

S1 Table. Germination metrics

	%dOD	t_max	%_max
**ZS6**	36.6 ± 1.12	54.0 ± 5.66	0.542 ± 0.0589
**ZS161**	39.8 ± 0.957	59.7 ± 2.87	0.665 ± 0.0422
**ZS180**	40.8 ± 1.31	48.7 ± 4.99	0.629 ± 0.0573
**ZS183**	46.3 ± 1.17	41.3 ± 3.3	0.976 ± 0.0451
**ZS456**	42.5 ± 0.787	52.7 ± 1.89	0.762 ± 0.0105
