# Supplementary material for: Engineering a probiotic Bacillus subtilis for acetaldehyde removal: A hag locus integration to robustly express acetaldehyde dehydrogenase
Source: PLoS One. 2024 Nov 7;19(11):e0312457. doi: 10.1371/journal.pone.0312457 (PMC11542774; doi:10.1371/journal.pone.0312457)
Supplement: S2 Table — (PDF) [file pone.0312457.s004.pdf]

S2 Table. Germination metrics in SIF

	%dOD	t_max	r_max
ZS161 LB AGK	30.2 ± 1.05	60.3 ± 10.8	0.377 ± 0.0919
ZS 161 SIF AGK	27.4 ± 2.43	63.3 ± 3.3	0.361 ± 0.0417
ZS183 LB AGK	36.4 ± 2.2	62.3 ± 5.91	0.597 ± 0.134
ZS 183 SIF AGK	34.0 ± 2.2	60.0 ± 3.56	0.516 ± 0.0516
